# The Effect of body Positive Social Media on Young Women with a History of Emotional Abuse and/or Bullying Victimization

**DOI:** 10.1007/s40653-026-00844-z

**Published:** 2026-02-27

**Authors:** Kirsty Martin, Myoungju Shin, Eva Kemps

**Affiliations:** 1 Charles Sturt University, Bathurst, Australia; 2 Flinders University, Adelaide, Australia

**Keywords:** Emotional abuse, Bullying, Body satisfaction, Body image, Body positivity, Social media

## Abstract

This study examined the effect of body-positive images from social media on mood and body satisfaction in young women who experienced childhood emotional abuse and/or bullying victimization compared to those without such adversities. A sample of 197 females aged 18–35 years was randomly assigned to view either body-positive, thin-ideal or neutral images, and rated their levels of mood and body satisfaction before and after viewing the images. Participants also completed questionnaires of emotional abuse/bullying victimization, body appreciation and self-objectification. Both emotional abuse and bullying victimization were associated with higher levels of negative mood (*r*s ≥ 0.190, *p*s ≤ 0.007), lower levels of positive mood (*r*s ≤ − 0.152, *p*s ≤ 0.033), body satisfaction (*r*s ≤ − 0.190, *p*s ≤ 0.008) and body appreciation (*r*s ≤ − 0.269, *p*s < 0.001), and greater self-objectification (*r*s ≥ 0.227, *p*s = 0.001). Importantly, body-positive images decreased negative mood, increased positive mood and body satisfaction, while thin-ideal images decreased mood and body satisfaction for both emotionally abused and not abused groups (*F*s = 10.91–23.07, *p*s < 0.001, η_p_^2^ = 0.105 – 0.199), and high and low bullied groups (*F*s = 9.76–17.24, *p*s < 0.001, η_p_^2^ = 0.095 – 0.156). Results suggest that body-positive images increase mood and body satisfaction in women with experiences of emotional abuse and/or bullying victimization in the same way as they do in women without such adversities. The findings add to the research on the effects of childhood trauma on mental health and body image and could inform interventions to reduce its negative impact.

## Introduction

Body satisfaction refers to the appraisal of one’s appearance and associated body image (Thompson et al., 1999). Many studies show that body satisfaction relates to an individual’s mental health and quality of life (e.g., Cash & Pruzinsky, 2002; Mond et al., 2013). High levels of body satisfaction are associated with overall greater psychosocial functioning (Davison & McCabe, 2006) and subjective well-being (Thompson et al., 1999). Conversely, low levels of body satisfaction have been associated with poor mental health, including low self-esteem (Frost & McKelvie, 2004), depressive mood (Pimenta et al., 2009), and health compromising behaviors such as smoking, drinking (King et al., 2005; Nelson et al., 2009), lack of exercise (More et al., 2019) and eating disorders (Matos et al., 2015; Neumark-Sztainer et al., 2006).

Although there are various risk factors associated with body dissatisfaction, studies suggest sociocultural pressure to be a key risk factor that contributes to body dissatisfaction (Frederick & Reynolds, 2022; Thompson et al., 1999). According to sociocultural theory, sociocultural sources (e.g., family, friends, the media) promote sociocultural beauty ideals (a thin and toned body is attractive and prestigious), especially in women, and exposure to these ideals contribute to body image concerns and a drive to attain these ideals (Thompson et al., 1999). Relatedly, objectification theory proposes that women in many cultures are valued for their appearance and sexuality, which leads them to consider these attributes as central aspects of their identity rather than their intellect, personality or desires (Frederick & Reynolds, 2022; Fredrickson & Roberts, 1997). Previous studies have shown that individuals who have experienced adversity in childhood, such as childhood maltreatment (Bodicker et al., 2022) (Vartanian et al., 2018) or childhood bullying (Day et al., 2022) are especially vulnerable to sociocultural pressures and body dissatisfaction.

Childhood maltreatment refers to exposure to negative life events such as physical, psychological/emotional and sexual abuse or neglect in childhood (Brown et al., 2009). All types of childhood maltreatment have been associated with negative mental health consequences, such as depression, anxiety, psychosis, suicidality, self-harm and substance abuse (see Baldwin et al., 2023 for a recent systematic review). In particular, emotional abuse (e.g., insults, shame, threats) in childhood has been suggested to cause damage to the developing brain, especially in the brain areas responsible for self-identity (Heim et al., 2013). Self-identity refers to one’s perception of oneself (e.g., self-awareness, self-evaluation) and is an important part of one’s social and emotional development (Adams & Marshall, 1996). Although it evolves throughout the lifespan, most of the development occurs in childhood and adolescence (a key developmental task for ages 11–18 years; Erikson, 1968). Parent-child interactions and parenting styles play an important role in the formation of self-identity (Erikson, 1968), and negative parent-child interactions contribute to the development of dysfunctional negative self-perception and self-schemas (Soffer et al., 2008). Furthermore, adversities such as abuse and maltreatment during childhood can disturb the normal development of self-identity (Bigras et al., 2015; Capella, 2017; Nielsen, 2016; Tafti et al., 2025; Vartanian et al., 2018). Identity disturbance is characterized by a persistent instability in self-perception and an incoherent self-concept (American Psychiatric Association, 2022), which lead to difficulties in defining personal values and goals (Jørgensen & Bøye, 2022). Consequently, those who lack a coherent sense of self and identity are more susceptible to sociocultural influences as they rely on external sources for self-definition (Campbell, 1990; Vartanian et al., 2018). Indeed, disruption in identity development has been associated with greater internalization of societal standards of attractiveness (Vartanian & Dey, 2013). Emotional abuse during childhood has been suggested to contribute to development of pathological self-criticism and distorted self-perception (Dunkley et al., 2010; Glassman et al., 2007), which are risk factors for body image dissatisfaction and eating disorders (Matos et al., 2015).

Similarly, studies also show that bullying victimization during childhood and adolescence is associated with shame, self-criticism and a sense of inferiority regarding one’s physical appearance and body image (See Day et al., 2022 for a systematic review). School bullying refers to intentional, repetitive, and aggressive behaviors such as verbal taunts, physical harm, and social exclusion, among school aged children and adolescents where the victim has less power than the perpetrator (Olweus, 1994). Body shape or weight (e.g., being over-weight or weight gain) is often the focus of victimization due to the sociocultural emphasis on attractiveness as an important value especially in women (Strahan et al., 2006). Being a victim of bullying has been associated with poor psychological wellbeing, including low self-esteem, higher levels of anxiety and depressive mood (deLara, 2019), and body image disturbance and body dissatisfaction, which persist into adulthood (Gattario & Frisen, 2019). Individuals who were victimized during childhood are also likely to report an objectified view of their body and to engage in constant surveillance of their appearance later in young adulthood (Lunde & Frisen, 2011). Interestingly, even victimization not related to body image can lead to body image disturbance as a way to reduce social threat (Day et al., 2022; Matos et al., 2015). As such, interventions that promote a more positive body image and ways to deal with the sociocultural emphasis on physical appearance (e.g., Gattario & Frisen, 2019) is important.

With increased access to mobile devices and the internet, social media (e.g., YouTube, Facebook, Instagram, TikTok) is ubiquitous in our day-to-day lives. It was reported that there are over 5 billion social media users worldwide in 2024 and this number is projected to increase to over 6 billion by 2028 (Statistica, 2025). Social media has been criticized for playing a significant role in promoting sociocultural beauty standards (Fioravanti et al., 2022; Holland & Tiggemann, 2016). Higher levels of social media usage and exposure to images of attractive celebrities and peers have been associated with upward social comparison (Fardouly & Vartanian, 2015), the tendency to compare oneself with others who are superior (Buunk & Gibbons, 2007), contributing to a decrease in body satisfaction and mood (Brown & Tiggemann, 2016; Fardouly & Vartanian, 2015). Recently however, a “body positive movement” or “body positivity” has emerged across social media. This movement aims to empower women to accept, appreciate and love their bodies, by providing a more realistic representation of the wider population, and challenging previously homogenous beauty standards (Cohen et al., 2021; Politte-Corn & Fardouly, 2020; Hendrickse et al., 2021). Body-positive social media images often include diverse body shapes and sizes, and what may be considered traditional imperfections such as stretch marks and cellulite (Cohen et al. 2019a, b b). Encouraging phrases or statements relating to accepting and loving one’s unique body are also included in many body-positive images (Cowels et al., 2023). These images that promote body positivity increase self-acceptance, through a broader conceptualization of beauty (Rodgers et al., 2023; Tylka & Wood-Barcalow, 2015). Several experimental studies have experimentally investigated the effects of exposure to body-positive images in comparison to thin-ideal or beauty-ideal images found on social media platforms. Improvements in body satisfaction and mood were reported among women after exposure to body-positive Instagram posts, in comparison to thin-ideal or beauty-ideal images (Cohen et al. 2019a, b a; Cowles et al., 2023; Hendrickse et al., 2021; Nelson et al., 2022; see Vandenbosch et al., 2022 for a recent review). It has been suggested that exposure to body-positive images increases body appreciation (Cohen et al. 2019a, b a). Positive body image has been linked to various well-being indices including self-compassion, self-esteem and positive emotions (Tylka, 2019). Further support for body positive social media comes from an ecological momentary assessment study (Stevens & Griffiths, 2020). In that study, participants reported their daily state body satisfaction, affective functioning and body positive image exposure. The results showed increased body satisfaction, decreased negative mood and increased positive mood after exposure to body positive social media.

The findings of improvements in mood and body satisfaction after viewing body positive images suggest that these images may benefit all social media users, particularly those who are more susceptible to the effects of appearance comparisons and self-objectification, such as individuals with a history of emotional abuse and school bullying (Emirtekin et al., 2019). To the authors’ knowledge, no research to date has considered the effect of body positive social media on individuals with a history of emotional abuse or bullying victimization as opposed to those who did not experience such adversities. Hence, research should address this gap in the literature by considering the impact of body-positive social media in this particularly vulnerable population.

Thus, the aim of the present study was to examine the effect of body positive images on levels of body satisfaction and mood in young women with a history of emotional abuse and/or bullying victimization in childhood and adolescence, compared with those who did not experience such adversities. As social comparison with celebrities and peers is shown to be greater among women than men (e.g., Ho et al., 2016), previous studies of body image and social comparison focused specifically on women (e.g., Brown & Tiggemann, 2020; Slater et al., 2019; Pedalino & Camerini, 2022). As such, the current study also focused on women. Similar to Cohen et al. ([Bibr CR15][Bibr CR16] a), participants completed questions on body satisfaction and mood before and after they viewed either body-positive images, thin-ideal images or neutral images. We expected two possible outcomes regarding the effect of body positive images in young women with a history of emotional abuse and/or bullying victimization. First, as individuals with a history of emotional abuse and/or bullying are more sensitive to beauty standards from sociocultural sources (e.g., peers, social media) (Vartanian & Dey, 2013; Gattario & Frisen, 2019), they may exhibit a greater increase in body satisfaction and mood after viewing social media images that promote body positivity than those without this adverse history. Second, conversely, individuals with a history of emotional abuse/bullying victimization report higher levels of negative mood and body dissatisfaction (Dye, 2020; Matos et al., 2015; Gattario & Frisen, 2019), which may dampen the positive effect of body positive social media for these individuals compared those without this adverse history.

## Methods

### Participants

Thirty participants (women, aged 18–35 years) completed a brief pilot study for the selection of the images (body positive, thin ideal) for the main study. Subsequently, 254 volunteers completed the main study. Inclusion criteria were identifying as a female and being 18–35 years of age. Exclusion criteria were identifying as other than female and being outside the 18–35 years age bracket. Of the 254 participants who completed the main survey, 28 were removed due to not meeting the inclusion criteria: not meeting the age criterion (*N* = 21) and identifying as having a biological sex other than female (*N* = 7). A further 29 participants were removed due to withdrawing consent at the end of the survey (*N* = 21) or exceeding a completion time of more than 4280 s (i.e., responses that are too slow, taking more than one minute per question/image, *N* = 8). The final sample consisted of 197 women aged 18–35 years (*M* = 24.66, *SD* = 3.95). G*Power analysis suggested that a sample size of 162 participants would yield a small to medium effect size with alpha 0.05 and 80% power, deeming the current sample to be adequate. Participants were recruited from the general public via snowball sampling, social media recruitment posts on Facebook, Instagram and LinkedIn (*N* = 174, 88%), and research participation pool of undergraduate psychology students at the first author’s university (*N* = 23, 12%). The psychology students received research participation time credit worth 15 min for participating in the main study. No other incentives were provided. All procedures were approved by the ethics committee at the first author’s university (H23634).

### Procedure

The Qualtrics online platform was used for both the pilot and the main studies. In the pilot study, after providing informed consent, participants completed demographic questions on age and gender. Participants then rated 50 body-positive and 50 thin-ideal images based on the provided definitions of “thin-ideal” and “body-positive”, using a visual analogue scale (VAS, 0 = *not at all*, 100 = *to a great extent*). The pilot study took approximately five minutes to complete.

In the main study, participants were told that the study investigated memory rather than body image to discourage them from paying too close attention to the body images viewed in the experimental conditions, and to reduce bias in responses (Cohen et al. 2019a, b a). After providing informed consent, participants first completed demographic questions on age, gender, height, weight, country of residence, ethnicity, and social media usage. Following this, participants completed the pre-state mood and body satisfaction VAS. Participants were then randomly allocated to one of the three conditions: body positivity (*N* = 65), thin-ideal (*N* = 56), or neutral (*N* = 61), and viewed 20 images specific to their assigned condition. Participants viewed each image for a minimum of 10 s after which they were able to move on to the next image by clicking a button. After viewing the images, participants completed the post-state mood and body satisfaction VAS, Body Appreciation Scale-2, and Objectified Body Consciousness Scale. Finally, participants completed the ACE study scale, and the 9-item bullying victimization subscale from the Revised Olweus Bully/Victim Questionnaire for Students. The study took approximately 15 min to complete. Participants were de-briefed at the conclusion of the study.

### Materials

#### Experimental task

##### ***Visual Stimuli***

A pilot study was conducted to select the thin-ideal and body-positive images from a collection of 50 per image type. Images were selected from public Instagram profiles, featuring celebrities or peers. Thin-ideal images were selected using the #victoriasecretmodels hashtag and body-positive social media images were selected using the #bodypositivity hashtag. Participants in the pilot study were provided with definitions for ‘thin-ideal’ (“These images are represented by thin, toned, attractive women, often representing idealized beauty standards. In thin-ideal Instagram posts, women are often wearing bikinis, figure-hugging or revealing clothing, or active-wear”) and ‘body-positive’ (“Promotes acceptance of all bodies, regardless of size, shape, or skin tone. Encouraging women to love and accept their bodies and rejecting unrealistic beauty standards. Body positivity Instagram posts often include women embracing their unique bodies and messages around body acceptance”). 20 images that were most highly rated to match the definition provided per condition were selected for use in the main study; thin-ideal (M = 83.73, SD = 8.16) and body positive (M = 84.16, SD = 3.70), resulting in a total of 40 images. 

Final image selection for the thin-ideal condition included close-up and full body images of women wearing swimwear (3), activewear (2), underwear (11), form-fitting clothes (2), and robes (2). Women depicted in the selected images were similar in age to the study sample. Final image selection for the body-positive condition included a variety of close-up and full body images of women wearing swimwear (6), activewear (1), underwear (4), form-fitting clothes (1), and cartoon-style images of women (8). Images depicted happy, strong, and proud women of varying sizes, shape and cultural backgrounds. Additionally, body positive quotes (referring to acceptance, diversity and beauty in all bodies), that had been included in the original Instagram posts, were included in nine images (as in Cowles et al., 2023). The cartoon-style images of women and body-positive quotes were included as they are representative of body-positivity on social media, thus enhancing ecological validity. The use of cartoon-style images of women and body-positive quotes is consistent with previous studies (Cohen et al. 2019a, b a; Cowles et al., 2023). Lastly, the visual stimuli included 20 appearance-neutral images that were selected using the #nature hashtag. Appearance-neutral images included wildlife, landscapes, seascapes, and flora.

#### Self-Report Measures

##### ***State Mood***

The negative mood scale included two questions asking about depressed and anxious feelings (e.g., ‘How anxious do you feel right now?’), whereas the positive mood scale included two questions referring to feeling confident and happy (e.g., ‘How happy do you feel right now?’) (Cohen et al. 2019a, b a). Participants responded to these questions using a Visual Analogue Scale (VAS, from ‘*not at all*’ (0) to ‘*very much*’ (100), prior to and after viewing the images in the experimental task (Cohen et al. 2019a, b a). The ratings for the two questions were averaged for each negative and positive mood scale in the study. Scores range from 0 to 100, with higher scores representing higher negative and positive mood states, respectively. In the study, mean scores for negative mood were 31.82 (24.94) before exposure to the images and 26.76 (22.26) after exposure to the images. Mean scores for positive mood before and after the images were 48.09 (22.90) and 51.97 (24.18), respectively. Cronbach’s alpha for the current sample was 0.76 for the two-item negative mood subscale before exposure to the images and 0.78 after the exposure. For positive mood subscale, Cronbach’s alpha was 0.80 for pre-exposure and 0.84 for post-exposure.

##### ***Body Satisfaction***

Three questions referring to satisfaction of weight, overall appearance, and body shape (e.g., ‘How satisfied are you with your body shape?’) provided a measure of state body satisfaction (Cowles et al., 2023). Similar to the state mood scales, participants responded to these questions using a Visual Analogue Scale (VAS), prior to and after viewing the images in the experimental task (Cohen et al. 2019a, b a). The ratings for the three questions were averaged in the study. Scores range from 0 to 100, with higher scores representing higher state body satisfaction. The mean scores for the sample were 44.59 (25.11) before and 47.39 (27.89) after exposure to the images. Cronbach’s alpha for the current sample was 0.87 for body satisfaction pre-exposure and 0.93 for post-exposure.

##### ***State Body Appreciation***

The Body Appreciation Scale-2 (BAS-2) was used to measure state body appreciation (Avalos et al., 2005). The BAS-2 addresses elements of love, acceptance, and respect for one’s body, rather than focusing on appearance (Tylka & Wood-Barcalow, 2015). Participants were provided with 10 statements such as ‘I respect my body’ and ‘I am comfortable in my body’. Participants were asked to rate how they felt about their body ‘right now’ on a 5-point Likert scale from ‘*Never*’ (1) to ‘*Always’* (5). Scores range from 10 to 50 with higher averaged scores indicating higher body appreciation. The mean score of the sample was 32.45 (9.18). Cronbach’s alpha for the current sample was .95. A principal component analysis on the current sample (KMO = 0.95, Bartlett’s test of sphericity, χ2 = 1752.59, df = 45, p < .001) revealed one factor with an eigenvalue greater than one, accounting for 70.25% of the variance.

##### ***State Self-Objectification***

An adapted version of the Objectified Body Consciousness Scale (OBCS) (McKinley & Hyde, 1996) was used to measure state self-objectification. The eight-item Body Surveillance subscale included statements such as ‘During the day, I think about how I look many times’. The eight-item Body Shame subscale included statements such as ‘I feel ashamed of myself when I haven’t made the effort to look my best’. Participants were asked to consider how they felt ‘right now’. Participants responded on a 7-point Likert scale from ‘*Strongly Disagree*’ (1) to ‘*Strongly Agree’* (7). Scores range from 16 to 112, with higher scores demonstrating higher levels of state body surveillance and body shame. The mean score for the sample was 67.07 (16.48). Cronbach’s alpha was 0.83 for the body surveillance subscale and 0.86 for the body shame subscale. A principal component analysis on the current sample (KMO = 0.880, Bartlett’s test of sphericity, χ2 = 1356.66, df = 120, p < .001) revealed a two-factor solution, accounting for 50.34% of the variance. The item loadings for each component were comparable to the loadings of McKinley and Hyde (1996).

##### ***Emotional Abuse***

The emotional abuse subscale of the Adverse Childhood Experiences (ACE) Study Scale (Felitti et al., 1998) was used to measure childhood history of emotional abuse. Participants were asked two polar questions (1 = ‘*yes*’ or ‘*no*’) relating to experiences prior to their 18th birthday (e.g., ‘Did a parent or other adult in the household often or very often swear at you, insult you, put you down, or humiliate you?’). Scores ranged from 0 to 2. Any ‘*yes*’ responses suggest experience of childhood emotional abuse. Following Felitti et al. (1998), participants who responded “*yes*” to any of the two items were classified as the emotionally abused group, and those who responded “*no*” to both items were classified as the not emotionally abused group. Cronbach’s alpha for the current sample was .72.

##### ***Bullying***

The bullying victimization subscale from the Revised Olweus Bully/Victim Questionnaire for Students (Olweus, 1996), was used to measure bullying as an element of childhood emotional abuse. Participants were provided with 10 statements such as ‘I was called mean names, made fun of, or teased in a hurtful way’. Questions were re-written in the past tense to evoke retrospective responses (Hoetger et al., 2015). Participants were asked to consider how often they had experienced each one prior to their 18th birthday, on a 5-point Likert scale from ‘*I did not experience this*’ (1) to ‘*I experienced this several times per week*’ (5). Scores were averaged and ranged from 10 to 50 with higher scores representing higher levels of bullying. Cronbach’s alpha for the current sample was .88. For the purpose of analyses, participants who scored below the average (*M* = 17.35, *SD* = 7.14) were classified as the low bullied group (*N* = 134) and those who scored above the average were classified as the high bullied group (*N* = 63), similar to previous studies which have used this measure as a dichotomous variable (e.g., Le et al., 2023; Rapee et al., 2020).

##### ***Social Media Usage***

The number of social media accessed per day (1-item) was measured using a 7-point Likert scale from ‘*Hardly ever’* (1) to ‘*More times than I can count’* (7). The mean score for the sample was 5.91 (1.51). Time spent on social media per day (1-item) was measured using a 13-point Likert scale from ‘*1–15 minutes*’ (1) to ‘*10 + hours*’ (13). The mean score for the sample was 5.24 (2.32). These measures replicate those used in previous studies (Cohen et al. 2019a, b a; Cowles et al., 2023).

##### ***Distractor questions***

Distractor questions relating to satisfaction with one’s social life, financial status, housing, and occupation (1 question each), were included to aid in concealing the purpose of the study. The answers to these questions were not analyzed in the study.

### Data Analysis

Correlational analyses were used to examine the relationships between social media use/emotional abuse/bullying victimization and other variables (body appreciation, state self-objectification, mood, body satisfaction). A series of one-way analysis of variance (ANOVA) were used to examine any pre-existing differences between conditions. A 2 (emotional abuse/bullying victimization: yes and no/high and low) × 3 (condition: body-positive, thin-ideal and neutral) × 2 (time: pre, post) mixed ANCOVA was then performed on negative and positive mood and body satisfaction, with BMI as a covariate, as it has previously been associated with body dissatisfaction in women (Austin et al., 2009; Frederick et al., 2007). This analysis examined the effects of emotional abuse/bullying victimization and condition on negative and positive mood and body satisfaction.

## Results

### Sample Characteristics

Sample characteristics are presented in Table 1. A majority of participants identified as Caucasian/White/European (70.6%), followed by east or southeast Asian (10.7%), and South Asian (8.6%). Mean self-reported body mass index (BMI, *M =* 24.75, SD = 5.72) fell under the healthy weight range (Centers for Disease Control and Prevention, 2024).

**Table 1:** Sample characteristics

	Emotional abuse		Bullying	
	Yes	No	High	Low
Age (years)	25.03	24.36	24.71	24.63
18–35 years	(4.24)	(3.70)	(4.27)	(3.80)
Gender (freq.)	87	110	63	134
Female				
BMI (kg/m2, mean/SD)	26.12 (6.13)	23.66 (5.14)	27.03 (6.84)	23.69 (4.79)
Negative mood (mean/SD)	34.53 (24.04)	25.15 (20.36)	35.53 (25.26)	26.36 (20.51)
Positive mood (mean/SD)	44.39 (20.55)	54.50 (21.29)	44.96 (19.68)	52.42 (21.99)
Body satisfaction (mean/SD)	38.30 (25.53)	52.07 (24.05)	39.16 (27.59)	49.21 (24.02)
Body appreciation (mean/SD)	28.26 (8.84)	35.75 (8.06)	28.35 (9.37)	34.37 (8.47)
State self-objectification (mean/SD)	71.57 (16.97)	63.50 (15.23)	72.70 (18.55)	64.42 (14.76)
Social media access per day (mean/SD)	5.79 (1.56)	6.00 (1.48)	5.98 (1.51)	5.87 (1.52)
Social media time spent per day (mean/SD)	5.47 (2.31)	5.06 (2.32)	5.84 (2.46)	4.96 (2.21)

The range of scores for the current sample was 16.41–51.99 for BMI, 0.00–95.75 for negative mood, 0.00–100.00 for positive mood, 0.33–100.00 for body satisfaction, 13.00–50.00 for body appreciation, 18.00–101.00 for self-objectification, 1.00–7.00 for the number of times social media was accessed per day, and 1.00–13.00 for the amount of time spent on social media per day

### Data Preparation and Correlational Analyses

As can be seen in Table 2, more time spent on social media was associated with lower body appreciation, higher state self-objectification, and greater negative mood. The number of social media accessed per day was not associated with body appreciation, state self-objectification, mood or body satisfaction. Both emotional abuse and bullying victimization correlated with lower body appreciation, higher state self-objectification, greater negative mood, lower positive mood and lower body satisfaction. Emotional abuse strongly correlated with bullying victimization.

**Table 2:** Pearson correlation coefficients for key variables

	SM (access)	SM (time)	Bullying	EA	BAS	OBCS	Negative mood	Positive mood
SM (access)	-							
SM (time)	**0.354**	-						
Bullying	0.031	**0.162**	-					
EA	− 0.094	0.106	**0.535**	-				
BAS	− 0.010	**− 0.305**	**− 0.269**	**− 0.384**	-			
OBCS	0.071	**0.250**	**0.227**	**0.228**	**− 0.633**	-		
Negative Mood	0.079	**0.228**	**0.190**	**0.205**	**− 0.477**	**− 0.460**	-	
Positive Mood	0.003	− 0.129	**− 0.152**	**− 0.219**	**0.566**	**0.399**	**− 0.504**	-
Body Satisfaction	0.099	− 0.130	**− 0.190**	**− 0.238**	**0.720**	**0.514**	**− 0.308**	**0.572**

Significant effects highlighted in bold. SM (access) = the number of times social media was accessed per day (range = 1.00–7.00); SM (time) = time spent on social media per day (range = 1.00–13.00); *EA* emotional abuse (range = 0.00–2.00); *BAS* body appreciation scale (range = 13.00–50.00); *OBCS* Objectified Body Consciousness Scale (range = 27.00–110.00); the range of scores for the current sample was 0.00–95.75 for negative mood, 0.00–100.00 for positive mood, 0.33–100.00 for body satisfaction and 10.00–41.00 for bullying

### Emotional Abuse, Condition and time

**Fig. 1:**
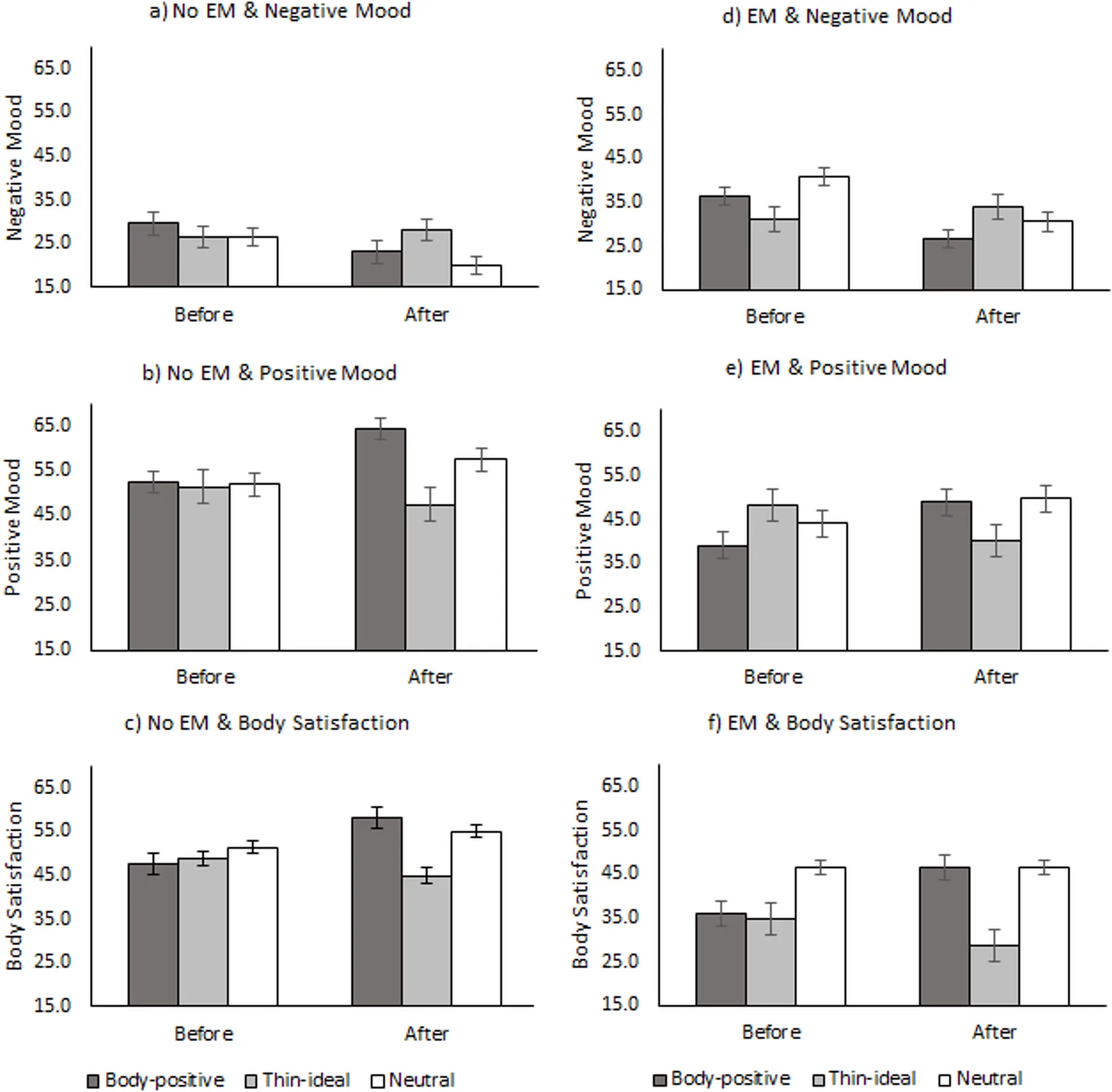
Effects of body-positive, thin-ideal and neutral images on mood and body satisfaction in women with and without a history of emotional abuse. Note. No EM = No emotional abuse; EM = Emotional abuse; Ranges for negative mood = 0 -95.75; Ranges for positive mood = 0 - 100; Ranges for body satisfaction = 0.33-100. Error bars reflect 95% confidence intervals

A series of one-way ANOVAs showed no statistically significant pre-existing differences between the condition groups (body-positive, thin-ideal and neutral) in terms of age, BMI, emotional abuse, bullying victimization, negative mood, positive mood and body satisfaction (all *F*s < 1.73, all *p*s > 0.180). There were significant interactions between condition and time for negative mood, positive mood and body satisfaction (Fig. 1). Compared with before viewing the images, negative mood decreased after viewing body-positive images and neutral images (both *p*s < 0.001), but did not change after viewing thin-ideal images (*p* = .260) (*F* (2,186) = 10.91, *p* < .001, η_p_^2^ = 0.105). Positive mood increased after viewing body-positive and neutral images (both *p*s < 0.014), but decreased after viewing thin-ideal images (*p* = .014) (*F* (2,186) = 13.37, *p* < .001, η_p_^2^ = 0.126). Body satisfaction increased after viewing body-positive images (*p* < .001), but decreased after viewing thin-ideal images (*p* = .004) (*F* (2,186) = 23.07, *p* < .001, η_p_^2^ = 0.199). Body satisfaction did not change after viewing neutral images (*p* = .232).

There was also a significant main effect of condition on body satisfaction (*F* (2,186) = 3.22, *p* = .042, η_p_^2^ = 0.033), but not on negative (*F* (2,186) = 0.03, *p* = .969) or positive mood (*F* (2,186) = 0.81, *p* = .45). Across pre- and post-exposure (i.e., on average), body satisfaction was lower in the thin-ideal image condition (*M* = 39.31, *S.E.* = 3.12) than in the neutral image condition (*M* = 49.85, *S.E.* = 2.91, *p* = .014), but it did not differ between the thin-ideal and body-positive image conditions, nor between the body-positive and neutral image conditions (all *p*s > 0.071). Finally, the main effect of time was not significant for negative mood, positive mood nor body satisfaction (all *F*s < 1.32, all *p*s > 0.252).

Interestingly, there was no three-way interaction between emotional abuse, condition and time (all *F*s < 0.518, all *p*s > 0.596), nor a two-way interaction between emotional abuse and condition (all *F*s < 0.778, all *p*s > 0.461), indicating that the effects of body-positive, thin-ideal and neutral images on negative and positive mood and body satisfaction did not differ between the emotionally abused and not abused groups. However, the main effect of emotional abuse was significant (mean standard errors are shown in Table 3): in comparison with the not emotionally abused group, the emotionally abused group showed greater negative mood (*F* (1,186) = 5.29, *p* = .023, η_p_^2^ = 0.028), lower positive mood (*F* (1,186) = 8.46, *p* = .004, η_p_^2^ = 0.043) and lower body satisfaction (*F* (1,186) = 10.13, *p* = .002, η_p_^2^ = 0.052).

**Table 3:** Negative mood, positive mood and body satisfaction in women with and without emotional abuse history

	Emotional abuse	No emotional abuse
Negative mood	M = 33.26 SD = 2.46 Range = 0–93.50	M = 25.65 SD = 2.15 Range = 0–95.75
Positive mood	M = 45.05 SD = 2.34 Range = 0–99.50	M = 54.21 SD = 2.05 Range = 1.50–100
Body satisfaction	M = 39.81 SD = 2.61 Range = 0.33–100	M = 50.96 SD = 2.28 Range = 1.17–100

### Bullying Victimization, Condition and time

The results were largely similar to those for emotional abuse, condition and time. Main or interaction effects involving bullying victimization are reported below in the main text (see Appendix A for full details). Similar to the findings for emotional abuse, there was no three-way interaction between bullying victimization, condition and time (all *F*s < 1.36, all *p*s > 0.260), nor a two-way interaction between bullying and condition (all *F*s < 1.57, all *p*s > 0.146), indicating that the effects of condition (body-positive, thin-ideal or neutral images) on negative mood, positive mood and body satisfaction did not differ between the low and high bullying victimization groups.

There was, however, a significant interaction between bullying victimization and time for negative mood (*F*(1,187) = 6.53, *p* = .011, η_p_^2^ = 0.034), but not for positive mood (*F* (1,187) = 0.005, *p* = .942) or body satisfaction (*F* (1,187) = 0.94, *p* = .333) (mean standard errors are shown in Table 4): both the low and high bullying victimization groups reported greater negative mood before viewing the images than after (all *p*s < 0.015). The high bullying victimization group reported greater negative mood than the low bullying victimization group before viewing the images (*p* = .008), but negative mood did not differ between the two groups after viewing the images (*p* = .183). The main effect of bullying was also significant, such that the high bullying victimization group reported greater negative mood (*F* (1,187) = 4.54, *p* = .034, η_p_^2^ = 0.024), marginally lower positive mood (*F* (1,187) = 3.36, *p* = .068) and lower body satisfaction (*F* (1,187) = 5.64, *p* = .019, η_p_^2^ = 0.029) than the low bullying victimization group.

**Table 4:** Negative mood, positive mood and body satisfaction in women with high and low bullying victimization history before and after viewing body image stimuli

	Time	High bullying victimization	Low bullying victimization
Negative mood	Before	M = 38.07 SD = 3.15 Range = 0–100	M = 27.92 SD = 2.09 Range = 0–100
	After	M = 29.62 SD = 2.87 Range = 0–87	M = 24.99 SD = 1.91 Range = 0–91.50
Positive mood	Before	M = 44.22 SD = 2.97 Range = 0–100	M = 50.47 SD = 1.98 Range = 0–100
	After	M = 48.12 SD = 3.07 Range = 0–100	M = 54.15 SD = 2.05 Range = 3–100
Body satisfaction	Before	M = 39.17 SD = 3.18 Range = 0–100	M = 47.41 SD = 2.12 Range = 0–100
	After	M = 40.63 SD = 3.47 Range = 0–100	M = 50.83 SD = 2.31 Range = 0–100

## Discussion

The aim of the present study was to examine, for the first time, the effect of body-positive images on mood and body satisfaction in young women with a history of emotional abuse and/or bullying victimization in childhood and adolescence. Results showed that body-positive images led to a decrease in negative mood, an increase in positive mood and an increase in body satisfaction. In contrast, exposure to thin-ideal images did not produce any changes in negative mood, but resulted in a decrease in positive mood and a decrease in body satisfaction. For neutral images, there was a decrease in negative mood, an increase in positive mood, but no changes in body satisfaction. Importantly, emotional abuse/bullying victimization had an overall negative effect on mood and body satisfaction. However, the effect of body-positive images (i.e., improvement in mood and body satisfaction) did not differ between those with and without a history of emotional abuse, and between those with low versus high levels of bullying victimization. That is, we observed the positive effect of body-positive images in participants with experiences of emotional abuse and/or bullying victimization similar to those without such experiences. Nevertheless, both emotional abuse and bullying victimization were associated with lower body appreciation and greater self-objectification.

The decrease in mood and body satisfaction post thin-ideal image exposure is consistent with previous experimental studies that examined negative effects of thin-ideal images on body image in women (e.g., Brown & Tiggemann, 2016; Cohen et al. 2019a, b a; Cowles et al., 2023). Increased availability and ownership of smartphones (Pew Research Center, 2024) means that anyone can easily view these thin-ideal images on social media. Although these images are not real representations of the wider population, frequent exposure and comparison to thin-ideal images of peers and celebrities on social media can lead to feelings of inferiority, lowered self-esteem, mood and body satisfaction (Brown & Tiggemann, 2016).

The increase in mood and body satisfaction after viewing body-positive images is consistent with recent studies (e.g., Cohen et al. 2019a, b a; Cowles et al., 2023; Rodgers et al., 2023; Vandenbosch et al., 2022) that showed similar results. The body-positive images in the current study were taken directly from social media and included various body shapes of women and encouraging statements that aimed to help women accept and appreciate their bodies. These body-positive images on social media show that different body shapes and looks are beautiful and that beauty includes inner qualities (e.g., confidence and self-acceptance) of a person as well as outer appearances (Rodgers et al., 2023; Tylka & Iannantuono, 2016). The finding of improvements in mood and body satisfaction after viewing body-positive images suggests that exposure to these body-positive images may help reduce the pervasive effects of thin-ideal images on women. However, the current study did not directly test this idea and further research is required to determine whether exposure to body-positive images can indeed counteract the harmful effects of thin-ideal images.

More importantly, although women with a history of emotional abuse and/or high levels of bullying victimization showed overall lower levels of mood and body satisfaction than those without a history of emotional abuse and/or with low levels of bullying victimization (in line with Dunkley et al., 2010; Puhl & Suh, 2015), both groups of women exhibited an increase in mood and body satisfaction after viewing body-positive images. This finding demonstrates that body-positive images have beneficial effects on body image in women regardless of any adverse history that negatively impacts mental health and body image. Although the current findings suggest that all women would benefit from body positive images on social media, it is possible that those who are susceptible to poor body image (such as individuals with a history of emotional abuse and/or bullying victimization) may benefit more from these images as their mood and body satisfaction levels are lower than others’. Low levels of body satisfaction have been linked to poor psychosocial functioning and subjective well-being (Thompson et al., 1999). Accordingly, exposure to body-positive images may lead to a greater improvement in psychosocial functioning and well-being in vulnerable populations whose body satisfaction is lower than those without such vulnerability. In addition, exposure to body-positive images on social media might be more effective in improving body satisfaction than a body image therapy session, for example, in these populations, as the exposure to these images on social media occurs more organically and also more frequently in real life.

As previous studies have shown that individuals with a history of emotional abuse and/or bullying are more sensitive to sociocultural influences (e.g., images on social media, Campbell, 1990; Lunde & Frisén, 2011; Thompson et al., 1999; Vartanian et al., 2018; Vartanian & Dey, 2013), we also considered the possibility that body positive images may have a greater effect on these individuals, leading to a greater increase in mood and body satisfaction than those without an adverse history. However, the current findings were not in line with this expectation. It may be that the brief exposure to the images (approximately 3–4 min in total) was not sufficiently long to elicit a greater increase in mood and body satisfaction. Alternatively, it is possible that individuals with a history of emotional abuse/bullying are more resistant to the beneficial effects of body-positive images, as the impact of the adverse history on body image may be hard to overcome. The current finding shows that this was not the case and that those with an adverse history also benefited from exposure to body-positive images.

Like most studies, the current study has a number of limitations. First, the body-positive images included both images of women of various body shapes and encouraging messages designed to help women accept and appreciate their bodies, because previous studies have shown that body images on their own do not have an effect on body satisfaction and that the inclusion of encouraging messages is necessary (Cowles et al., 2023). Moreover, the meaning intended by these images might not be clear to some people without the messages, as the intended meaning (e.g., all body shapes can be beautiful) is different from the prevailing expectation of sociocultural beauty standards. Nevertheless, the inclusion of both body images of women and encouraging messages makes it difficult to determine whether the effect of body-positive images is from the body images or the encouraging messages included in these images. Second, participants completed the questions about body satisfaction immediately after viewing the body-positive images. Although the observed increase in body satisfaction is encouraging, especially among women who have experienced emotional abuse/bullying, the longevity and stability of this effect remain to be determined. Thus, future longitudinal research is needed to ascertain any longer-term effects of exposure to body-positive images on body satisfaction. Third, the present study focused only on emotional abuse, rather than all subtypes of childhood maltreatment. We did so as previous studies have shown that individuals with a history of emotional abuse experience poorer mental health compared to those who experienced other types of abuse (e.g., Dye, 2020). Nevertheless, as different types of child maltreatment often co-occur (Dong et al., 2004), the inclusion of other types of maltreatment (e.g., physical abuse, sexual abuse, neglect) in future studies could helpfully investigate the effect of body-positive images in women who experienced a wider range of child maltreatment. Lastly, similar to previous studies of body image and social comparison (Brown & Tiggemann, 2020; Slater et al., 2019; Pedalino & Camerini, 2022), the present study focused solely on women, because social comparison with celebrities and peers has been shown to be greater in women than in men (Ho et al., 2016). However, future research could usefully investigate whether the effects of body-positive images on body satisfaction and mood would similarly occur in other genders.

In conclusion, the present study examined the effect of body-positive images on mood and body satisfaction in young women with a history of emotional abuse and/or bullying victimization. Exposure to body-positive images increased mood and body satisfaction in all women regardless of any adverse history that negatively impacted their mental health and body image. The findings add to the research on the effects of childhood trauma on mental health and body image and could inform interventions to reduce its negative impact.

## Data Availability

The data for the study can be found at https://data.mendeley.com/datasets/2vnz2ng6hp/2.

## Appendix A

**Table 5:** Results from a 2 (Bullying: Bullying, No-bullying) x 3 (Condition: Body-positive, Thin-ideal, Neutral) x 2 (Time: Before, After) Mixed ANCOVA with BMI as a covariate: Main and interaction effects on negative mood, positive mood and body satisfaction

Factors	Main or Interaction effects		
	Negative mood	Positive mood	Body satisfaction
Bullying	***F*** **(1**,**187) = 4.54**, ***p*** **= .034**,** η**_**p**_^**2**^ **= 0.024**	*F* (1,187) = 3.36, *p* = .068	***F*** **(1**,**187) = 5.64**, ***p*** **= .019**,** η**_**p**_^**2**^ **= 0.029**
Condition	*F* (2,187) = 0.13, *p* = .882	*F* (2,187) = 0.30, *p* = .739	***F*** **(2**,**187) = 3.36**, ***p*** **= .037**,** η**_**p**_^**2**^ **= 0.035**
Time	*F* (1,187) = 2.60, *p* = .109, η_p_^2^ = 0.014	*F* (1,187) = 1.49, *p* = .223	*F* (1,187) = 0.04, *p* = .846
Bullying × Condition	*F* (2,187) = 1.95, *p* = .146	*F* (2,187) = 1.76, *p* = .174	*F* (2,187) = 1.57, *p* = .211
Bullying × Time	***F*****(1**,**187) = 6.53**, ***p*** **= .011**,** η**_**p**_^**2**^ **= 0.034**	*F* (1,187) = 0.005, *p* = .942	*F* (1,187) = 0.94, *p* = .333
Condition × Time	***F*****(2**,**187) = 9.76**, ***p*** **< .001**,** η**_**p**_^**2**^ **= 0.095**	***F*****(2**,**187) = 9.88**, ***p*** **< .001**,** η**_**p**_^**2**^ **= 0.096**	***F*** **(2**,**187) = 17.24**, ***p*** **< .001**,** η**_**p**_^**2**^ **= 0.156**
Bullying × Condition × Time	*F* (2,191) = 1.36, *p* = .260	*F* (2,187) = 0.159, *p* = .853	*F* (2,187) = 0.60, *p* = .552

*BMI* body mass index. BMI was used as a covariate. The main effects of BMI on negative mood and positive moods were not significant, *F*s ≤ 2.31, *p*s ≥ 0.130. However, there was a significant main effect of BMI on body satisfaction, *F* (1,187) = 5.43, *p* = .021, η_p_^2^ = 0.028. The interaction effects between BMI and time on negative mood, positive moods and body satisfaction were not significant, *F*s ≤ 0.14, *p*s ≥ 0.445
